# Multispectral photoacoustic microscopy based on an optical–acoustic objective

**DOI:** 10.1016/j.pacs.2014.12.004

**Published:** 2015-01-13

**Authors:** Rui Cao, Joseph P. Kilroy, Bo Ning, Tianxiong Wang, John A. Hossack, Song Hu

**Affiliations:** Department of Biomedical Engineering, University of Virginia, Charlottesville, VA 22908, United States

**Keywords:** Photoacoustic microscopy, Optical–acoustic objective, Multispectral imaging, Cell nucleus, Blood vessel, Lipid

## Abstract

We have developed reflection-mode multispectral photoacoustic microscopy (PAM) based on a novel optical–acoustic objective that integrates a customized ultrasonic transducer and a commercial reflective microscope objective into one solid piece. This technical innovation provides zero chromatic aberration and convenient confocal alignment of the optical excitation and acoustic detection. With a wavelength-tunable optical-parametric-oscillator laser, we have demonstrated multispectral PAM over an ultrabroad spectral range of 270–1300 nm. A near-constant lateral resolution of ∼2.8 μm is achieved experimentally. Capitalizing on the consistent performance over the ultraviolet, visible, and near-infrared range, multispectral PAM enables label-free concurrent imaging of cell nucleus (DNA/RNA contrast at 270 nm), blood vessel (hemoglobin contrast at 532 nm), and sebaceous gland (lipid contrast at 1260 nm) at the same spatial scale in a living mouse ear.

## 1 Introduction

Photoacoustic microscopy (PAM) [1–3] fills the long-standing gap in high-resolution imaging of endogenous optical absorption contrasts *in vivo*, among which DNA/RNA [4], hemoglobin [5], and lipid [6,7] are of particular interest. Specifically, the cell nucleus is a critical organelle containing DNA genome, which strongly absorbs the ultraviolet light. Morphological changes in cell nuclei, including enlargement and envelope folding, are considered hallmarks of cancer cells [8]. Hemoglobin, a dominant absorber in the visible spectral range, is the primary oxygen carrier in the blood circulation. Angiogenesis [9,10] and hypoxia [11], which can be respectively revealed by the distribution and oxygen saturation of hemoglobin, are also core hallmarks of cancer [12]. Lipid forms a diverse group of infrared-absorbing molecules that play important roles at cellular and organismal levels [13]. Aberrant lipid metabolism is an established hallmark of cancer cells [14]. Concurrent imaging of the multiple endogenous optical absorbers at the same spatial scale holds great promise for both basic and translational cancer research. However, multispectral PAM that spans from ultraviolet to near-infrared is complicated by the chromatic aberration of the optics and not currently available.

To address this unmet challenge, we have developed a novel multispectral PAM system. Specifically, a commercial reflective microscope objective with zero chromatic aberration is employed to achieve consistent optical focusing over a broad spectral range. A customized ultrasonic transducer is attached to the dark zone of the reflective objective for convenient confocal alignment of the optical excitation and acoustic detection in reflection mode, which avoids the optical aberration and acoustic loss induced by the otherwise needed optical–acoustic beam combiners in conventional PAM systems [1,2,15,16]. The ring-shaped transducer design provides an alternative means for the confocal alignment without a combiner, but at the expense of detection sensitivity due to the central opening [17,18]. Transmission-mode PAM [19,20] can be readily extended for multispectral measurements by using the aberration-free reflective objective; however, its application is limited by the poor accessibility to anatomical sites *in vivo*.

With a high-repetition-rate wavelength-tunable optical parametric oscillator (OPO) laser, our multispectral PAM covers an unprecedented spectral range of 270–1300 nm. A near-constant lateral resolution of ∼2.8 μm is achieved experimentally. Capitalizing on the ultrabroad spectral coverage and consistent spatial resolution, we have demonstrated concurrent PAM of cell nuclei (DNA/RNA contrast at 270 nm), blood vessel (hemoglobin contrast at 532 nm), and sebaceous gland (lipid contrast at 1260 nm) at the same spatial scale in a living mouse ear.

## 2 Materials and methods

### 2.1 Optical–acoustic objective

**Fig. 1:**
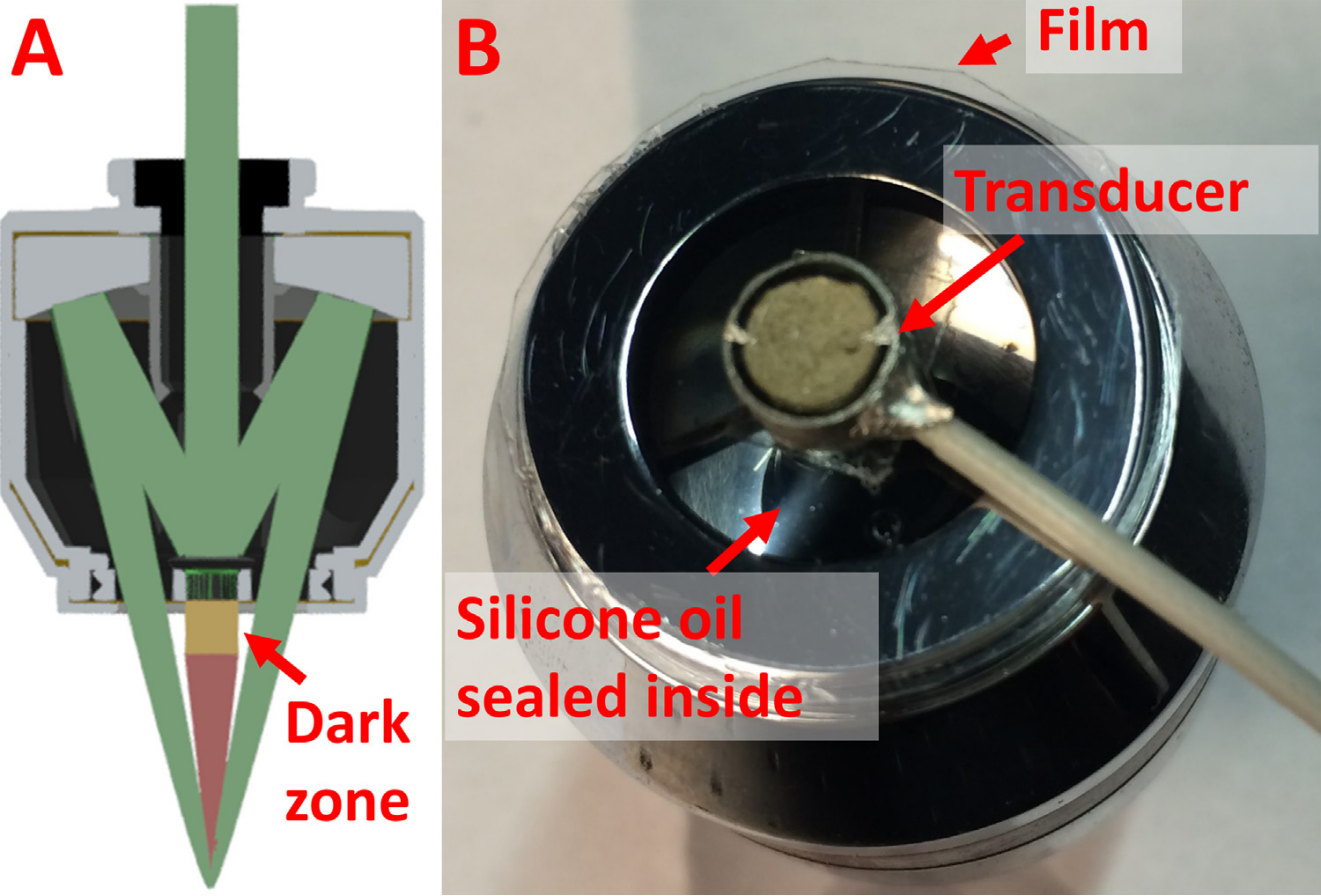
(A) Sectional view of the optical–acoustic objective. The yellow block represents the ultrasonic transducer. Optical and acoustic paths are labeled in green and red, respectively. (B) Photograph of the objective showing the liquid-filling feature.

As shown in Fig. 1A, the optical–acoustic objective consists of a commercial reflective microscope objective (also known as Schwarzschild objective; LMM-15X-UVV, Thorlabs) and a customized ultrasonic transducer. The two hemispherical mirrors of the reflective microscope objective are coated with ultraviolet-enhanced aluminum, which assures zero chromatic aberration over a broad spectral range (200 nm–20 μm). The convex primary mirror causes an obscuration in the center of the imaging system. Thus, the laser beam through this reflective objective is solid only at the focus and donut-shaped everywhere else. The ultrasonic transducer is attached to the back surface of the primary mirror, which is directly below the entrance pupil of the reflective microscope objective. Positioning the transducer in the optically dark zone allows convenient alignment of the optical and acoustic foci with no interference. To ensure optimal superposition of the optical and acoustic foci, the focal length of the piezoelectric ceramic piston transducer is carefully designed and the transducer location fine tuned before permanent attachment to the reflective microscope objective. The elegant confocal alignment and easy coupling of acoustic energy to the transducer make the optical–acoustic objective ideally suited for reflection-mode PAM.

For acoustic coupling, the optical–acoustic objective needs to be immersed in a transparent liquid. The mismatch of optical refractive index at the interface between the liquid and the air cavity of the objective induces significant optical aberration [21], particularly during mechanical scan when the liquid surface is unstable. To address this issue, the objective is filled with the same liquid and sealed with a thin film (OCA8146-2, Thorlabs), which is optically transparent over 270–2000 nm (Fig. 1B). Silicone oil, the liquid we use, is non-absorbing and commonly used in oil-immersion microscope objective (*e.g.* UPLSAPO30XSIR, Olympus). To avoid the lensing effect induced by the surface tension of silicone oil, the entrance pupil of the objective is also sealed with a fused-silica broadband optical window (WG41050, Thorlabs). This liquid-filling feature clearly distinguishes our design from the previous PAM system based on a reflective microscope objective [21].

### 2.2 Multispectral PAM system

**Fig. 2:**
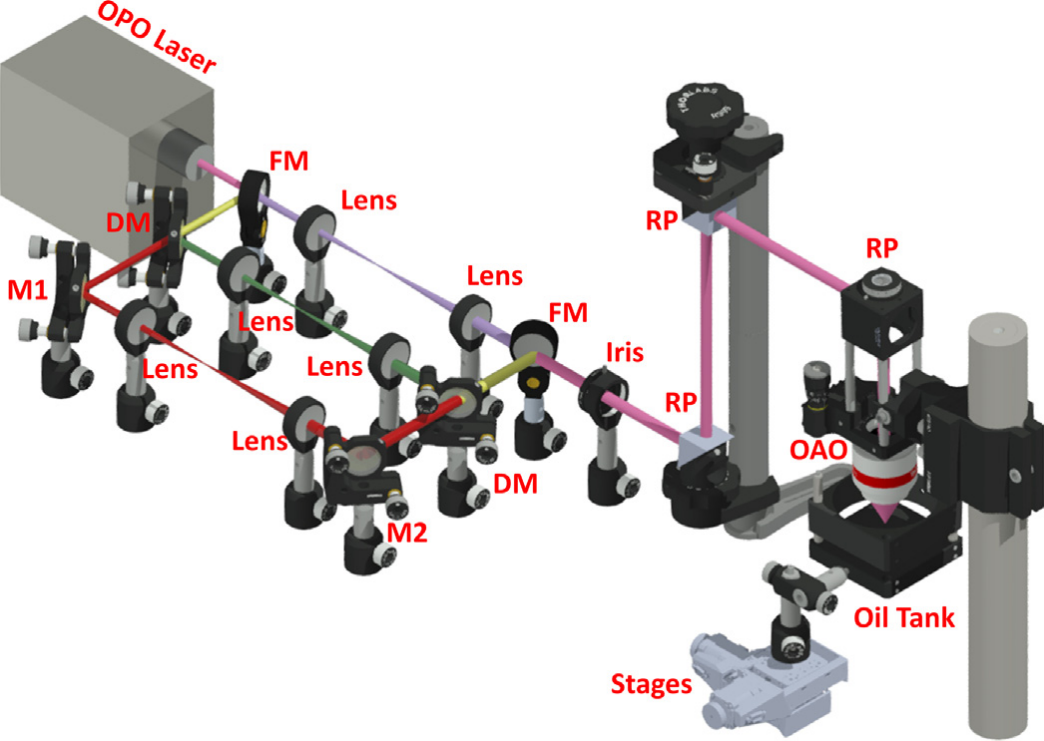
Schematic of multispectral PAM. The ultraviolet, visible, and near-infrared paths are labeled in purple, green, and red, respectively. The visible and near-infrared combined path is labeled in yellow. The combined path of all three spectral ranges is labeled in pink. OPO, optical parametric oscillator; FM, flip mirror; DM, dichroic mirror; M1 and M2, mirrors; RP, right-angle prism; OAO, optical–acoustic objective.

As shown in Fig. 2, our multispectral PAM system employs a wavelength-tunable OPO laser (NT242, Ekspla; wavelength coverage: 210–2600 nm; repetition rate: 1 kHz). Due to the non-uniform beam shape across the broad spectral range, the laser output is split by a flip mirror (FM; TRF90, Thorlabs) and a dichroic mirror (DM; DMLP650, Thorlabs) into three paths—ultraviolet (purple), visible (green), and near-infrared (red)—for beam reshaping. The individually reshaped and expanded beams are combined *via* another identical pair of FM and DM, spatially filtered by an iris with an 8-mm aperture (ID25, Thorlabs), reflected by three fused-silica broadband right-angle prisms (RP; PS611, Thorlabs), and focused by the optical–acoustic objective for multispectral photoacoustic excitation. The ratios of the pulse energies after and before beam reshaping and filtering are 80.9%, 64.3%, and 60.8% for the ultraviolet, visible, and near-infrared paths, respectively. The objective is immersed in an oil tank filled with silicone oil for acoustic coupling. The bottom of tank is sealed with a thin layer of transparent polyethylene membrane to expose the object to be imaged. Commercial ultrasound gel (Aquasonic CLEAR^®^, Parker Laboratories) is sandwiched between the membrane and object for acoustic coupling. The oil tank and object holder are mounted on two motorized linear stages (PLS-85, PI micos) for two-dimensional raster scanning.

## 3 Results

### 3.1 Performance of the optical–acoustic objective

**Fig. 3:**
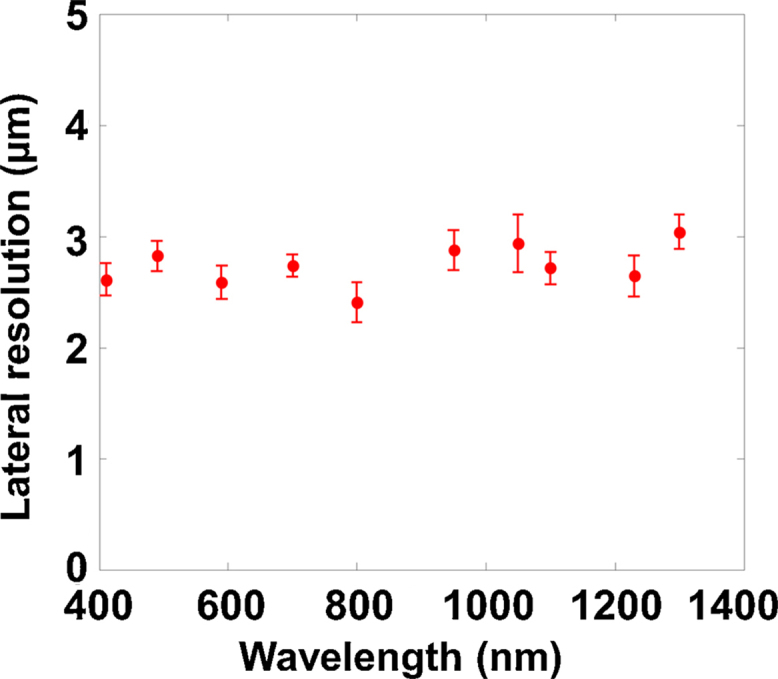
Near-constant lateral resolution of multispectral PAM over a broad spectral range.

Optical focusing of the optical–acoustic objective was evaluated by PAM of a 7-μm carbon fiber (S-CF706-T700, CST). To test its potential for multispectral imaging, the photoacoustic excitation wavelength was swept from 210 nm to 1400 nm with a spectral interval of ∼100 nm. No photoacoustic signal of the carbon fiber was detected below 400 nm or above 1300 nm, probably due to the weak optical absorption of carbon fiber and/or the low laser energy at these spectral ranges. At each selected wavelength within the detectable range, a cross section of the fiber was repeatedly scanned 50 times for statistics. The mean values and standard errors of the measured diameter of the optical focus were shown in Fig. 3. Over the broad spectral range of 400–1300 nm, zero chromatic aberration results in a near-constant optical focal diameter of ∼2.8 μm, which makes the optical–acoustic objective ideal for concurrent PAM of multiple endogenous absorbers at the same spatial scale *in vivo*.

**Fig. 4:**
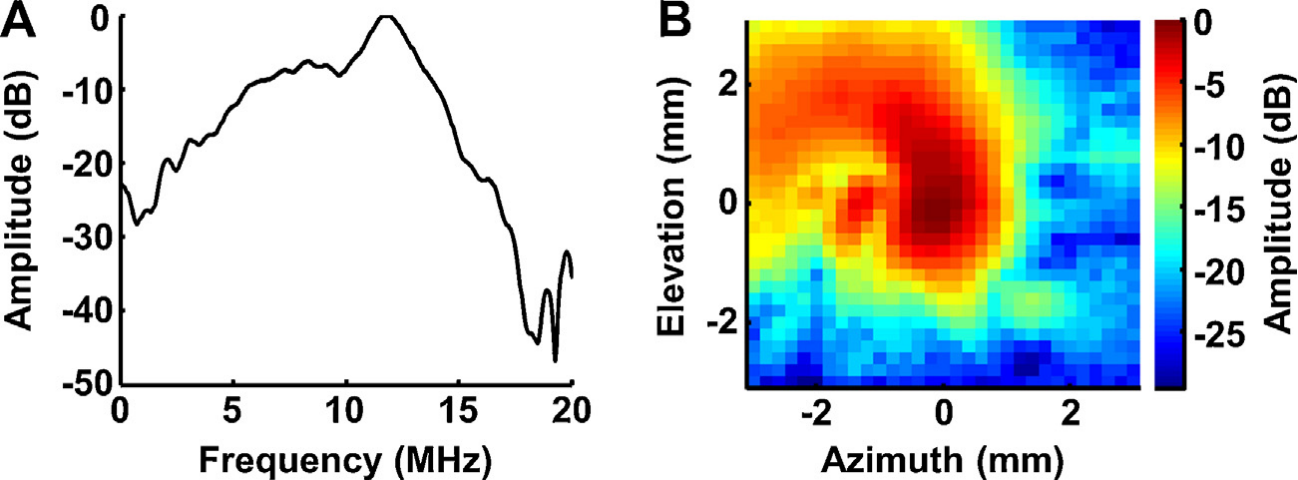
(A) Frequency response and (B) transverse acoustic-detection field of the customized ultrasonic transducer.

The acoustic performance of the optical–acoustic objective was also evaluated. The frequency response of the customized transducer, characterized using an ultrasonic pulser-receiver (5900PR, Panametrics), shows a central frequency of ∼11.8 MHz and a 6-dB bandwidth of 24% (Fig. 4A). The acoustic-detection field of the transducer was scanned using a commercial hydrophone transducer (HGL-0085, ONDA; bandwidth: 0.5 kHz–40 MHz) motorized by a three-dimensional linear stage. The 6-dB acoustic beamwidth along the azimuthal and elevational directions are 1.5 mm and 2.5 mm, respectively (Fig. 4B). The asymmetric field distribution is due to imperfect fabrication. The distance between the acoustic focal plane and back surface of transducer is ∼24 mm, well matching the working distance of reflective microscope objective for the confocal alignment of the optical and acoustic foci.

### 3.2 Multispectral PAM *in vivo*

The *in vivo* performance of the multispectral PAM was tested in the ear of a nude mouse (Crl:NU-Foxn1^nu^, Charles River Laboratories; 6-month old). Throughout the experiment, the mouse was maintained under anesthesia with 1.2% vaporized isoflurane and the body temperature was set at 37 °C using a heating pad. All experimental animal procedures were carried out in conformity with the laboratory animal protocol approved by the Animal Care and Use Committee at the University of Virginia.

**Fig. 5:**
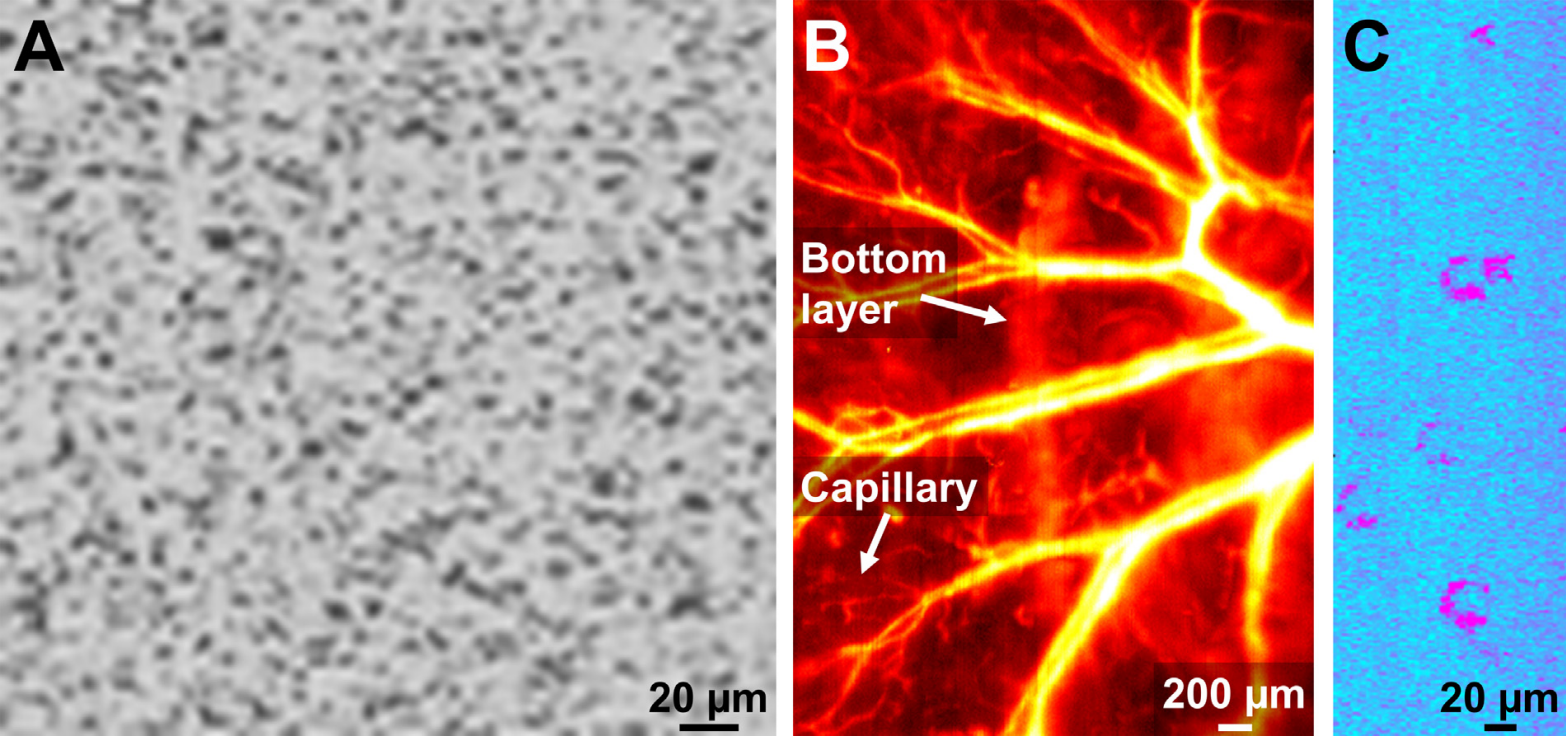
Multispectral PAM of (A) cell nucleus (270 nm), (B) blood vessel (532 nm), and (C) sebaceous gland (1260 nm) in the mouse ear *in vivo*.

Three major endogenous optical absorbers—cell nucleus, blood hemoglobin, and lipid—were imaged using our multispectral PAM with ultraviolet, visible, and near-infrared excitations, respectively (Fig. 5). Specifically, the cell nucleus was imaged at 270 nm (Fig. 5A), where two major cellular components—DNA and RNA—have high optical absorption [22]. The imaged cell nuclei show a uniform distribution with an average diameter of ∼6 μm, which is in agreement with the previous report [22]. The vascular anatomy was imaged at 532 nm (Fig. 5B), an isosbestic wavelength of hemoglobin where oxy- and deoxy-hemoglobin absorb light equally. With the 2.8-μm lateral resolution, the ear vasculature down to single capillaries was resolved. Operating at a relatively low acoustic frequency, the multispectral PAM imaged both the top and bottom vascular layers of the ear. The sebaceous gland was imaged at 1260 nm (Fig. 5C), where the lipid absorption peaks [23]. The unique U shape of sebaceous glands was clearly observed. The relatively low optical absorption of silicone oil at the wavelength range 1200–1300 nm [24] showed negligible attenuation to the laser excitation.

The A-line rate of our system is currently limited to 1 kHz by the OPO laser. In order to restrict the scan time, we used different step sizes to image regions of interest with different dimensions. Specifically, the spatial intervals between adjacent A-lines and B-scans were respectively set to 5/6 μm and 5/3 μm for imaging the cell nucleus, 5/2 μm and 5 μm for imaging the blood vessel, and 5/2 μm and 20/3 μm for imaging the sebaceous gland. The total scan time for the ear vasculature (4.5 mm × 3 mm) was 18 min.

## 4 Discussion

In this short communication, we reported multispectral PAM based on a novel optical–acoustic objective. By integrating a customized ultrasonic transducer and a commercial reflective microscope objective, our optical–acoustical objective features four unique capabilities:

• Acoustic detection in the optically dark zone enables elegant optical–acoustic confocal alignment and produces no interference between the optical excitation and acoustic detection. Compared with existing optical–acoustic combining strategies in our first-generation [1], second-generation [2], and fully motorized PAM [18], the optical–acoustic objective excels in low optical aberration and acoustic loss.
• Liquid filling minimizes optical aberration. For acoustic coupling, the optical–acoustic objective must be immersed in a transparent liquid. Filling the objective cavity with silicone oil eliminates the optical aberration induced by the refractive-index mismatch at the oil–cavity interface.
• Zero chromatic aberration provides consistent optical resolution over a broad spectral range. This feature makes our objective ideal for multispectral measurements.
• Fusing the ultrasonic transducer and optical objective into one solid piece allows non-degradable PAM performance without routine confocal alignment of the optical and acoustic foci as required for traditional PAM. In addition, the objective has a standard microscope thread that can be easily adopted for convenient integration of PAM and other mainstream optical microscopy techniques, including but not limited to optical coherence tomography, confocal microscopy, and multiphoton microscopy.

The axial resolution and sensitivity of multispectral PAM can be further improved by designing and fabricating a higher-frequency transducer with tighter acoustic focus. This improvement will enable the detection and differentiation of endogenous molecules with weak absorbance or overlapping absorption spectra, including but not limited to water [25], glucose [26], oxy- and deoxy-hemoglobin [5], and melanin [27]. Combining the multiple endogenous absorption contrasts may provide new insights into the pathogenic mechanisms of a broad spectrum of vascular and metabolic disorders at the cellular level.

## Conflict of interest

The authors declare that there are no conflicts of interest.
